# Health-related quality of life and distress in elderly vs. younger patients with high-grade glioma—results of a multicenter study

**DOI:** 10.1007/s00520-020-05354-8

**Published:** 2020-02-14

**Authors:** Mirjam Renovanz, Anne-Katrin Hickmann, Minou Nadji-Ohl, Naureen Keric, Elke Weimann, Christian Rainer Wirtz, Susanne Singer, Florian Ringel, Jan Coburger

**Affiliations:** 1 Department of Neurosurgery, University Medical Center, Mainz, Germany; 2 Division of Neuro-Oncology, University Medical Center Tubingen, Tübingen, Germany; 3 Department of Neurosurgery, Kantonsspital St. Gallen, St. Gallen, Switzerland; 4 Department of Neurosurgery, Klinikum Stuttgart, Stuttgart, Germany; 5 Department of Neurology, RKH Kliniken Ludwigsburg, Ludwigsburg, Germany; 6 Department of Neurosurgery, University Medical Center Ulm, Ulm, Germany; 7 Division of Epidemiology and Health Services Research, Institute of Medical Biostatistics, Epidemiology and Informatics, University Medical Center, Mainz, Germany

**Keywords:** Elderly patients, High-grade glioma, Glioma, Glioblastoma, Quality of life, Age, Karnofsky performance status, Distress

## Abstract

**Objective:**

Half of all newly diagnosed patients with glioblastoma are > 65 years still with a poor prognosis. Preserving quality of life is of high importance. However, patient reported outcome (PRO) data in this patient group is rare. The aim was to compare health-related quality of life (HRQoL) and distress between elderly and younger patients with high-grade glioma (HGG).

**Methods:**

We used baseline data of a prospective study where HGG patients were enrolled from 4 hospitals. Distress was measured using the distress thermometer (DT), HRQoL using the European Organization for Research and Treatment of Cancer (EORTC) Quality of Life Core Questionnaire (QLQ-C30) plus brain module (BN20). We compared distress and HRQoL by age (≥ 65 vs. < 65 years), gender, performance score, and time since diagnosis using multivariate linear and logistic regressions.

**Results:**

A total of *n* = 93 (30%) out of *n* = 309 patients were ≥ 65 years (mean 70 years, range 65–86 years). Mean DT score of elderly patients (5.2, SD 2.6) was comparable with younger patients (4.9, SD 2.6). Elderly patients reported significantly lower global health (GHS, mean elderly vs. younger; 50.8 vs. 60.5, *p* = 0.003), worse physical (56.8 vs. 73.3, *p* < 0.001) and lower cognitive functioning (51.1 vs. 63.2, *p* = 0.002), worse fatigue (52.5 vs. 43.5, *p* = 0.042), and worse motor dysfunction (34.9 vs. 23.6, *p* = 0.030). KPS and not age was consistently associated with HRQoL.

**Conclusion:**

Physical functioning was significantly reduced in the elderly compared with younger HGG patients, and at the same time, emotional functioning and DT scores were comparable. KPS shows a greater association with HRQoL than with calendric age in HGG patients reflecting the particular importance for adequate assessment of HRQoL and general condition in elderly patients.

## Introduction

High-grade gliomas (HGG) represent the majority of gliomas with glioblastomas showing an incidence of 3.19 (3.16–3.21) cases per 100,000 person years [1]. Half of all newly diagnosed patients with glioblastoma (GBM) are older than 65 years [2]. Although recently improved and cost-effective treatment schemes for elderly patients with GBM have been published, these patients are facing a particularly dismal prognosis with a median survival of less than 6 months [3–6] compared with younger healthier patients with more favorable outcomes and molecular marker profiles according to recent studies [2]. In many clinical trials, higher age is an exclusion criterion, and historically, many elderly patients received no tumor-specific therapy but best supportive care [7, 8]. Due to general condition (frailty) and comorbidities, treating elderly patients with HGG is challenging, and multidisciplinary approaches are required [9–11].

Therefore, preserving quality of life in elderly patients is of high importance particularly considering the short life expectancy. Distress and supportive care needs in HGG patients are high. They should be addressed early in the disease trajectory by psycho-oncologists but also supportive and palliative care services [12]. Additionally, quality of life during survival is equally important as the length of survival in the elderly population [12, 13]. Furthermore, it has been reported that lower performance status, higher age, female gender, and shorter time since diagnosis can be associated with distress and reduced quality of life [5]. However, health-related quality of life (HRQoL) data is rare for elderly people with HGG, and as soon as they experience clinical decline due to disease progression, assessment becomes difficult [2, 5].

Application of patient-reported outcomes (PROs) has become essential in assessing patients’ HRQoL, distress, and psychosocial burden, as well as supportive care needs [14]. Recently, it has been shown that monitoring symptoms via PRO measures can be very helpful for cancer patients and even influence survival [15–17]. As the elderly population in HGG in most of the studies is defined by an age ≥ 65 years, HRQoL and distress in this population might be different than in younger patients below than 65 years. Furthermore, so far, no data focusing especially on elderly patients’ HRQoL are available. Thus, a comparison might help clinicians in clinical daily practice to advise patients regarding this important issue.

Therefore, the aims of our study were to describe quality of life and distress in elderly patients with HGG and to compare the results with those of the younger ones focusing in psychological and physical issues. Furthermore, we aimed to investigate factors associated with HRQoL of HGG patients.

## Patients and method

### Patients

**Fig. 1:**
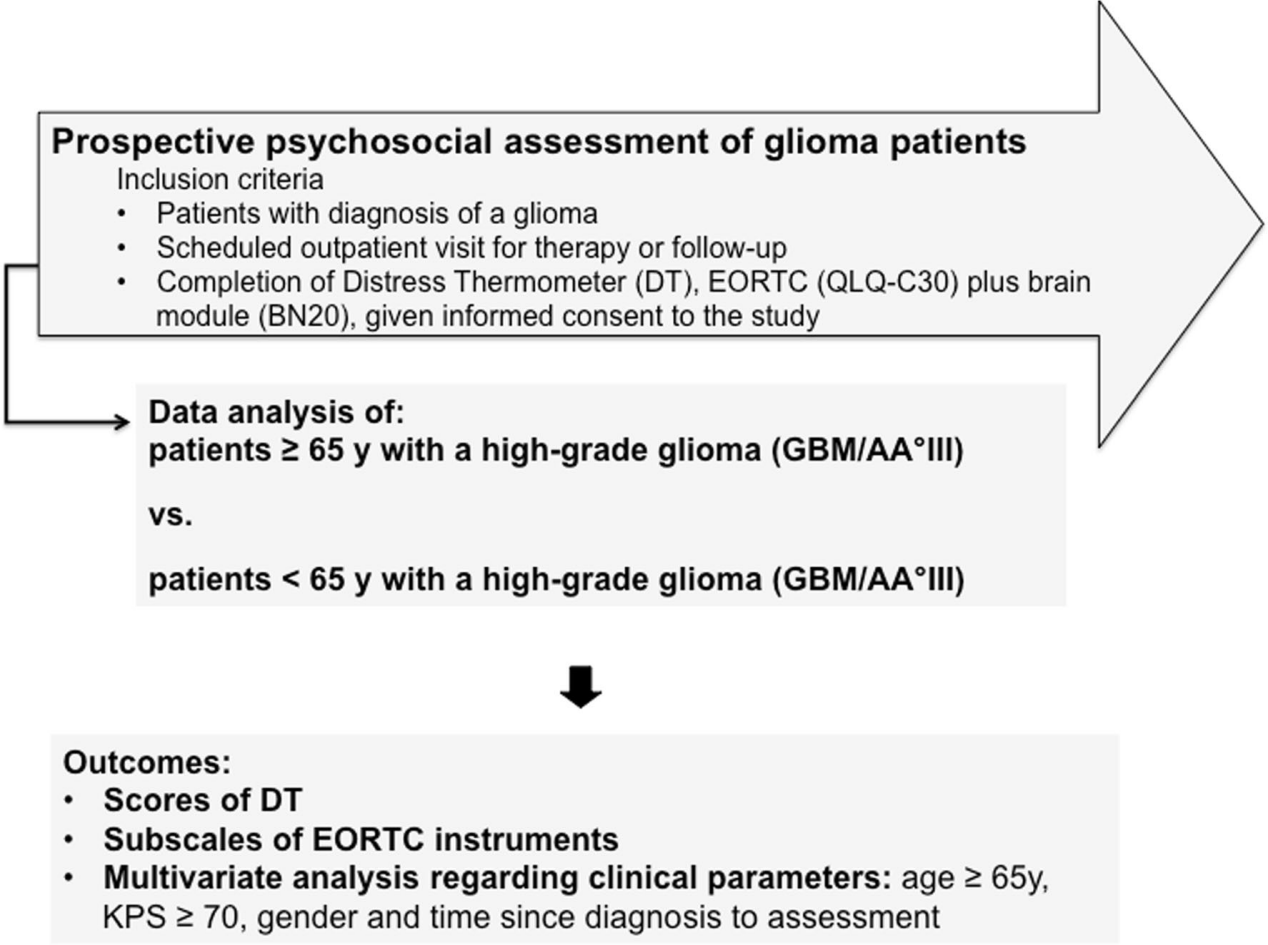
The course of the study

From March 2014 to October 2014 as well as April 2015 to June 2016, we conducted two prospective studies assessing HRQoL, distress, and supportive care needs in glioma patients. Patients at four German neuro-oncological centers were approached during their outpatient visits and asked to participate in the study as previously described [18–20]. Inclusion criteria data analysis was a diagnosis of glioma WHO grades III–IV regardless of disease stage (initial diagnosis or recurrent disease), absence of aphasia impairing communication or consent to the study, and given informed consent. Patients were asked to complete several PRO measures. Furthermore, demographic and clinical data were recorded in a database. If patients were assessed several times during the study, we only evaluated the first assessment per patient for this cross-sectional analysis. Figure 1 shows the course of the study. When patients declined the participation, gender, age, diagnosis, and if possible reason were documented.

### Instruments

#### Distress thermometer

The distress thermometer (DT) is a self-reporting screening instrument developed by the National Comprehensive Cancer Network to evaluate psychological distress on a visual analog scale (0–10 points). A problem list with 40 items is included for patients to indicate the area of concern (family, financial, and physical). Studies have proven its acceptance in oncological patients, and Goebel and colleagues evaluated the German version for brain tumor patients. A score ≥ 6 indicates a significant burden in brain tumor patients according to Goebel et al. [21].

#### European Organization for Research and Treatment of Cancer quality of life core questionnaire accompanied by the brain-specific module

The EORTC QLQ-C30 is a widely accepted questionnaire evaluating cancer patients’ quality of life. Functions’ symptom and the global health status (GHS) are investigated (physical (physf), role (rolef), emotional (emof), social (socf), and cognitive functioning (cogf), fatigue, nausea and vomiting, pain, dyspnea, insomnia, appetite loss, constipation, diarrhea, and financial difficulties). Its validity and reliability have been proven in numerous clinical studies, and it is available in 103 languages. The additional module for brain tumor patients (BN20) consists of 20 questions specifically assessing their symptoms (3 neurological deficit scales, 1 future uncertainty scale, treatment, and disease-related symptoms) [22, 23]. The scores were calculated according to the user manual. Each scale ranges from 0 to 100, with higher scores indicating better functioning for functional scales and worse symptoms for symptom scales.

### Statistical analysis

We performed a descriptive explorative assessment comparing scores of DT and subscales of EORTC instruments between the elderly and the younger group. The following parameters were considered to be potentially associated with distress and quality of life: performance status (KPS) ≥ 70, age (≥ 65 years, according to clinical studies), gender, and time since diagnosis [5, 24]. These variables were selected content driven by clinical relevance. We tested their association with univariate and multivariate models using binary logistic regression for distress (DT ≥ 6) and linear regression for quality of life (all EORTC subscales).

### Ethics

The study was in accordance with national law, institutional ethical standards, and the 1964 Helsinki Declaration and its later amendments. The ethic committees of all study centers approved the study (Mainz, Ludwigsburg, Stuttgart, and Ulm/Gunzburg, Germany, No: 837.349.15 (10117), and 837.472.13 (9157-F)). Patients provided written informed consent prior to data acquisition.

## Results

### Patients

A total of *n* = 93 (30%) out of *n* = 309 patients were ≥ 65 years (mean = 70 years, range 65–86 years). The majority of the elderly population harbored a glioblastoma (GBM, *n* = 77, 83%), *n* = 53 (57%) were male, median KPS was 70 (range 40–100), and mean time since diagnosis was 2.2 months. The younger population had a mean age of 48 years (range 19–64), *n* = 126 were male (57%), less patients had a GBM (*n* = 103, 47%), and the median KPS was 80 (range 40–100). Details are provided in Table 1.

**Table 1:** Clinical and demographic data of the patient sample. All variables are indicated in numbers and % in brackets if not otherwise specified

Variable	All patients *n* = 309 (100%)	Older patients *n* = 95 (100%)	Younger patients *n* = 214 (100%)
Age in years			
Mean (SD)	55 (14)	70 (4.5)	48 (10)
Median (range)	55 (19–86)	69 (65–86)	50 (19–64)
Gender			
Male	179 (58)	54 (57)	127 (59)
Female	130 (42)	41 (43)	87 (41)
Living situation			
Single	66 (21)	16 (17)	48 (23)
In relationship	229 (74)	76 (80)	155 (72)
Missing	14 (5)	3 (3)	11 (5)
Working situation			
Working	170 (55)	11 (12)	140 (65)
Retired or early retired	93 (30)	68 (71)	44 (21)
Missing	46 (15)	16 (17)	30 (14)
WHO grade			
WHO III	128 (41)	17 (18)	110 (51)
WHO IV	181 (59)	78 (82)	104 (49)
Survival at assessment			
< 1 year	128 (41)	52 (55)	76 (36)
> 1 and < 2 years	47 (15)	17 (18)	30 (14)
> 2 years	134 (43)	26 (27)	108 (50)
Tumor localization			
Frontal	128 (41)	37 (39)	91 (43)
Temporal	88 (28)	28 (29)	60 (28)
Parietal	21 (7)	7 (7)	14 (6)
Occipital	48 (16)	16 (18)	32 (15)
Other unknown	20 (7) 4 (1)	7 (7) 0	13 (6) 4 (2)
MGMT promotor methylation			
Unmethylated	128 (41)	46 (48)	82 (38)
Methylated	86 (28)	32 (34)	54 (26)
Not analyzed	73 (24)	14 (15)	59 (28)
Missing	22 (7)	3 (3)	19 (8)
IDH status			
IDH wild type	127 (41)	56 (59)	71 (33)
IDH mutated	84 (27)	20 (21)	64 (30)
Not analyzed	76 (25)	16 (17)	60 (28)
Missing	22 (7)	3 (3)	19 (9)
Ongoing chemotherapy			
Yes	125 (40)	53 (56)	72 (34)
No	158 (51)	39 (41)	119 (56)
Missing	26 (9)	3 (3)	23 (10)
Surgery for recurrent tumor			
Yes	87 (28)	14 (15)	73 (34)
No	188 (61)	67 (71)	121 (57)
Missing	34 (11)	14 (15)	20 (8)
Karnofsky index			
Mean (SD)	78 (16)	74 (18)	81 (15)
Median (range)	80 (30–100)	70 (40–100)	80 (30–100)
Time since diagnosis in months			
Mean (SD)	42 (51)	25 (44)	49 (54)
Median (range)	19 (0.5–298)	10 (0.5–288)	31 (0.5–296)

### Distress and health-related quality of life

Mean DT scores of elderly patients were comparable with those of younger patients (mean elderly patients vs. younger patients; 5.2 (SD = 2.6) vs. 4.9 (SD = 2.6), *p* = 0.42).

Elderly patients reported significantly lower GHS (mean elderly vs. younger; 50.8 vs. 60.5, *p* = 0.003), lower physf (56.8 vs. 73.3, *p* < 0.001), lower cogf (51.1 vs. 63.2, *p* = 0.002), higher fatigue (52.5 vs. 43.5, *p* = 0.042), greater impairment by visual disorders (23.9 vs.15.0, *p* = 0.013), by motor dysfunction (34.9 vs. 23.6, *p* = 0.030), by weakness of legs (31.4 vs. 20.8, *p* = 0.03), and greater problems regarding bladder control (22.3 vs. 10.8, *p* = 0.001). Younger patients reported significantly higher symptom scores for insomnia (elderly patients vs. younger patients; 27.0 vs. 36.4, *p* = 0.003) and financial difficulties (18.8 vs. 33.8, *p* < 0.001), please see Table 2.

**Table 2:** Comparison between patients ≥ 65 and < 65 years regarding score on DT and subscale or symptom scale on EORTC instruments

Score on distress thermometer resp. subscale or symptom scale on EORTC instruments	≥ 65 years mean (SD)	< 65 years mean (SD)	*p*
Distress score	5.2 (2.6)	4.9 (2.6)	0.42
Global health status (GHS)	50.8 (22.1)	60.5 (22.6)	0.003
Physical functioning	56.8 (33.6)	73.3 (27.1)	< 0.0001
Role functioning	52.6 (38.8)	60.1 (34.4)	0.21
Emotional functioning	62.3 (26.6)	60.0 (26.5)	0.66
Social functioning	52.5 (35.4)	57.6 (34.8)	0.35
Cognitive functioning	51.1 (33.8)	63.2 (31.4)	0.002
Fatigue	52.5 (30.6)	43.5 (29.3)	0.042
Nausea	7.7 (17.4)	8.3 (16.5)	0.49
Pain	24.9 (30.8)	24.2 (29.8)	0.702
Insomnia	27.0 (32.1)	36.4 (35.2)	0.003
Appetite loss	18.7 (28.8)	19.2 (30.3)	0.701
Constipation	18.2 (29.9)	15.5 (27.6)	0.69
Financial difficulties	18.8 (29.1)	33.8 (37.5)	< 0.0001
Future uncertainty	45.3 (30.4)	42.5 (29.2)	0.58
Visual disorder	23.9 (26.1)	15.0 (20.8)	0.013
Motor dysfunction	34.9 (31.5)	23.6 (25.7)	0.030
Communication deficit	35.7 (33.8)	28.4 (29.8)	0.40
Headaches	24.6 (33.3)	30.4 (33.9)	0.06
Seizures	5.4 (34.6)	8.5 (23.0)	0.20
Drowsiness	48.9 (34.6)	42.9 (31.6)	0.57
Itchy Skin	23.1 (30.1)	16.3 (26.2)	0.25
Hair loss	19.7 (32.2)	16.2 (31.1)	0.38
Weakness of legs	31.4 (36.1)	20.8 (31.3)	0.03
Bladder control	22.3 (33.8)	10.8 (25.3)	0.001

### Factors associated with distress and quality of life

In general, KPS was consistently associated with HRQoL regarding all functioning scales (physical functioning, *p* < 0.0001, 95% CI 30.73, 47.35; emotional functioning, *p* < 0.0001, 95% CI 9.29, 25.59; cognitive functioning, *p* < 0.0001, 95% CI 13.18, 33.72; social functioning, *p* < 0.0001, 95% CI 24.14, 45.48). This was true for most of the symptom scales as well as single item scales. Age same or above 65 years was associated with worse physical functioning (*p* = 0.029, 95% CI − 15.66, − 0.84), less financial difficulties (*p* < 0.0001, 95% CI − 29.99, − 9.36), visual disorders (*p* = 0.028, 95% CI 0.77, 13.48), and seizures (*p* = 0.030, 95% CI − 14.52, − 0.75). Details are provided in Table 3.

**Table 3:** Results of the multivariate analysis regarding the EORTC subscales and single items with content-driven-selected variables

Subscale or symptom	Variable	Beta	*p*	95% CI lower	95% CI upper
Physf	Age ≥ 65 (yes/no)	− 0.126	0.029	− 15.66	− 0.84
	Time since diagnosis	0.040	0.484	− 0.53	1.12
	KPS ≥ 70 (yes/no)	0.526	< 0.0001	30.73	47.35
	gender	− 0.060	0.278	− 10.58	3.05
Rolef	Age ≥ 65 (yes/no)	0.015	0.815	− 15.66	− 0.84
	Time since diagnosis	0.113	0.072	− .53	1.12
	KPS ≥ 70 (yes/no)	0.416	< 0.0001	30.73	47.35
	Gender	− 0.033	0.592	− 10.58	3.05
Emof	Age ≥ 65 (yes/no)	0.102	0.130	− 1.65	12.82
	Time since diagnosis	0.030	0.650	− 0.63	1.00
	KPS ≥ 70 (yes/no)	0.278	< 0.0001	9.29	25.59
	Gender	− 0.175	0.007	− 15.86	− 2.51
Cogf	Age ≥ 65 (yes/no)	− 0.084	0.205	− 15.01	3.24
	Time since diagnosis	0.071	0.280	− 0.47	1.60
	KPS ≥ 70 (yes/no)	0.294	< 0.0001	13.18	33.72
	Gender	− 0.080	0.213	− 13.75	3.08
Socf	Age ≥ 65 (yes/no)	0.011	0.863	− 8.60	10.24
	Time since diagnosis	0.083	0.191	− 0.37	1.77
	KPS ≥ 70 (yes/no)	0.408	< 0.0001	24.14	45.48
	Gender	− 0.065	0.294	− 13.32	4.05
Fatigue	Age ≥ 65 (yes/no)	0.057	0.391	− 4.70	11.97
	Time since diagnosis	− 0.094	0.147	− 1.61	0.24
	KPS ≥ 70 (yes/no)	− 0.291	< 0.0001	− 30.51	− 11.83
	Gender	0.120	0.061	− 0.34	14.99
Nausea	Age ≥ 65 (yes/no)	− 0.060	0.388	− 7.19	2.81
	Time since diagnosis	0.014	0.840	− 0.50	0.61
	KPS ≥ 70 (yes/no)	− 0.021	0.757	− 6.49	4.73
	Gender	0.156	0.021	0.84	10.01
Pain	Age ≥ 65 (yes/no)	0.028	0.690	− 6.93	10.45
	Time since diagnosis	0.082	0.226	− 0.37	1.56
	KPS ≥ 70 (yes/no)	− 0.133	0.053	− 19.38	0.120
	Gender	0.098	0.143	− 2.03	13.92
Dyspnea	Age ≥ 65 (yes/no)	0.087	0.212	− 3.16	14.15
	Time since diagnosis	− 0.044	0.519	− 1.28	0.65
	KPS ≥ 70 (yes/no)	− 0.134	0.050	− 19.39	0.02
	Gender	0.029	0.669	− 6.23	9.69
Insomnia	Age ≥ 65 (yes/no)	− 0.125	0.074	− 18.96	0.88
	Time since diagnosis	− 0.019	0.785	− 1.25	0.95
	KPS ≥ 70 (yes/no)	− 0.077	0.264	− 17.44	4.80
	Gender	0.123	0.069	− 0.65	17.59
Appetite loss	Age ≥ 65 (yes/no)	− 0.114	0.093	− 16.87	1.31
	Time since diagnosis	− 0.130	0.053	− 2.01	0.01
	KPS ≥ 70 (yes/no)	− 0.210	0.002	− 26.47	− 6.08
	Gender	0.114	0.082	− 0.95	15.73
Constipation	Age ≥ 65 (yes/no)	0.022	0.754	− 7.29	10.04
	Time since diagnosis	− 0.006	0.935	− 1.02	0.94
	KPS ≥ 70 (yes/no)	− 0.092	0.185	− 16.35	3.17
	Gender	0.106	0.117	− 1.61	14.35
Diarrhea	Age ≥ 65 (yes/no)	− 0.074	0.296	− 9.41	2.88
	Time since diagnosis	− 0.058	0.399	− 0.99	0.40
	KPS ≥ 70 (yes/no)	− 0.065	0.349	− 10.29	3.65
	Gender	0.011	0.868	− 5.17	6.13
Financial difficulties	Age ≥ 65 (yes/no)	− 0.257	< 0.0001	− 29.95	− 9.36
	Time since diagnosis	− 0.055	0.416	− 1.64	0.68
	KPS ≥ 70 (yes/no)	− 0.140	0.038	− 23.91	− 0.66
	Gender	− 0.040	0.542	− 12.42	6.55
GHS	Age ≥ 65 (yes/no)	− 0.125	0.058	− 12.28	0.202
	Time since diagnosis	0.003	0.960	− 0.69	0.72
	KPS ≥ 70 (yes/no)	0.327	< 0.0001	11.07	25.19
	Gender	− 0.022	0.726	− 6.78	4.73
Future uncertainty	Age ≥ 65 (yes/no)	− 0.072	0.287	− 12.95	3.85
	Time since diagnosis	− 0.162	0.015	− 2.07	− 0.22
	KPS ≥ 70 (yes/no)	− 0.238	< 0.0001	− 26.59	− 7.68
	Gender	0.018	0.780	− 6.60	8.78
Visual disorder	Age ≥ 65 (yes/no)	0.149	0.028	0.77	13.48
	Time since diagnosis	− 0.048	0.465	− 0.96	0.44
	KPS ≥ 70 (yes/no)	− 0.221	0.001	− 19.30	− 4.94
	Gender	0.058	0.373	− 3.18	8.45
Motor dysfunction	Age ≥ 65 (yes/no)	0.059	0.335	− 3.74	10.95
	Time since diagnosis	0.004	0.943	− 0.78	0.84
	KPS ≥ 70 (yes/no)	− 0.493	< 0.0001	− 42.60	− 26.13
	Gender	0.046	0.435	− 4.07	9.41
Communication deficit	Age ≥ 65 (yes/no)	− 0.001	0.990	− 8.88	8.76
	Time since diagnosis	0.012	0.858	− 0.89	1.06
	KPS ≥ 70 (yes/no)	− 0.356	< 0.0001	− 37.21	− 17.45
	Gender	0.000	0.999	− 8.08	8.07
Headache	Age ≥ 65 (yes/no)	− 0.082	0.238	− 15.76	3.94
	Time since diagnosis	0.045	0.508	− 0.72	1.45
	KPS ≥ 70 (yes/no)	− 0.021	0.760	− 12.77	9.33
	Gender	0.163	0.015	2.17	20.22
Seizures	Age ≥ 65 (yes/no)	− 0.151	0.030	− 14.52	− 0.75
	Time since diagnosis	0.021	0.755	− 0.63	0.87
	KPS ≥ 70 (yes/no)	− 0.205	0.003	− 19.36	− 4.01
	Gender	− 0.040	0.545	− 8.22	4.36
Drowsiness	Age ≥ 65 (yes/no)	0.060	0.378	− 5.12	13.42
	Time since diagnosis	0.020	0.761	− 0.87	1.18
	KPS ≥ 70 (yes/no)	− 0.255	< 0.0001	− 30.39	− 9.60
	Gender	0.012	0.850	− 7.68	9.32
Itchy skin	Age ≥ 65 (yes/no)	0.093	0.185	− 2.75	14.11
	Time since diagnosis	0.023	0.740	− 0.78	1.10
	KPS ≥ 70 (yes/no)	− 0.111	0.109	− 17.17	1.73
	Gender	0.079	0.242	− 3.14	12.35
Hair loss	Age ≥ 65 (yes/no)	0.020	0.768	− 7.85	10.61
	Time since diagnosis	0.070	0.290	− 0.47	1.56
	KPS ≥ 70 (yes/no)	− 0.042	0.535	− 13.69	7.13
	Gender	0.291	< 0.0001	20.71	27.64
Weakness of legs	Age ≥ 65 (yes/no)	0.062	0.360	− 5.23	14.33
	Time since diagnosis	− 0.053	0.426	− 1.51	0.64
	KPS ≥ 70 (yes/no)	− 0.307	< 0.0001	− 36.83	− 14.88
	Gender	0.038	0.557	− 6.25	11.56
Bladder control	Age ≥ 65 (yes/no)	0.129	0.062	− 0.43	17.32
	Time since diagnosis	0.032	0.638	− 0.74	1.21
	KPS ≥ 70 (yes/no)	− 0.210	0.002	− 25.75	− 5.74
	Gender	− 0.013	0.841	− 8.95	7.30

## Discussion

In our analysis comparing elderly and younger HGG patients, we found that the elderly population seems to be more affected by the disease than younger ones with regard to physical impairment. We found significant differences in scores between the two patient groups possibly highlighting the frailty of many elderly patients. However, regarding emotional function and DT, the results were comparable in both groups.

### Study population and generalizability of the data

Although this is one of the first studies describing HRQoL in HGG with a focus on age, we have to take into account that according to a former drop-out analysis of the study, 30% of all patients seen within the screening period declined the assessment [25]. Most of the decliners harbored a GBM and were in a significantly worse general condition; age was not found to be associated with study decline. Therefore, we probably present positively biased data for the elderly, but also for the younger population due to the selection bias of the study. Furthermore, we observed significant differences between the younger and the older patient group regarding WHO grade resulting in a higher percentage of patients in the younger patient group with a longer survival at the assessment than the older ones. However, it has been shown that DT and HRQoL are not influenced by WHO grade, and even in patients with meningioma, a significant burden can be observed [26–29]. Due to its greater incidence with higher age, GBM is diagnosed in older people more frequently than in the younger population [1]. Similarly, there were more patients under chemotherapy in the older patient group (56 vs. 34% in the younger patient group). This should be considered influencing the HRQoL.

### Results of younger and elderly patients with HGG

Both patient groups reported relatively low social and emotional functioning compared with the normal population reflecting the significance of the disease and the relevance of the topic [30]. In comparison with other cancer diagnoses, where younger patients are more distressed than elderly patients in general, our findings further emphasize how much more burdening a brain tumor diagnosis is for all age groups (REFs). Further, we found a significant association between longer time since diagnosis and lower future uncertainty in the regression analysis underlining the initial shock of the diagnosis and a certain adaption to the situation and the symptoms over time.

Elderly patients’ HRQoL was more affected than the younger one’s regarding physical impairment and motor dysfunction. However, emotional functioning was comparable. This is interesting and probably due to accentuated neurocognitive deficits in older patients, which permit full recognition of the severity of the situation. On the other hand, older patients may be more satisfied with their life lived thus far and less anxious about life-time lost due to the disease. Furthermore, elderly people could hesitate to allow depressive thoughts and try to present themselves strong, compliant, and positive in order to preserve their strength but also the support of the doctor as long as advanced care planning is not early enough provided [31, 32].

Yet, elderly patients reported significantly lower GHS, physical and cognitive functioning, greater fatigue, and impairment due to visual disorders as well as greater motor dysfunction meaning that they definitely perceived critical impairment, even though some may not be disease related. Although HRQoL assessed by the EORTC instruments is a subjective estimation, it remains sometimes unclear what the item or function does mean for the individual patient. Elderly patients may recognize their problems well but seem emotionally not as burdened as we would probably assume. Assessing quality of life by structured questionnaires like the EORTC instruments is a standardized approach where an individual weighting of issues is not intended in order to facilitate inter-individual comparisons. However, the assessment with individual consideration of the variety of topics influencing HRQoL (individualized approach) is complex, hard to implement in clinical routine or even in studies, and data are challenging to compare between patients [33–35]. However, regarding the brain tumor patient population, it may be worth to develop alternative approaches in order to assess the meaning of HRQoL for different individuals, e.g., by developing instruments more focusing on their situation or signaling questions implementable into clinical routine in order to facilitate, refine, and individualize the assessment.

In line with results of emotional functioning, the DT was comparable between the two groups. Finally, to admit a psychological burden may be more difficult than a physical impairment. Nonetheless, since some of the deficits older patients experience, such as loss in motor function, visual disturbances, weakness of legs, and loss of bladder control, may not be solely disease related and already persistent for a longer period of time, patients may have gotten used to them and find them less worrisome. Additionally, losing bodily functions may be more acceptable when growing old, in general. Greater fatigue in older patients may explain lower rates of insomnia compared with younger patients. On the other hand, cancer patients can develop symptoms of fatigue and present with sleeping disorder at the same time, which occur in brain tumor patients frequently and are suboptimal detected and treated by the neuro-oncologists [36].

Younger patients reported more often financial difficulties than elderly people, which is a significant problem in HGG patients. They often lose their higher executive functions and are not able to work anymore as they used to before the disease, leading to financial restrictions with significant consequences for their family and caregivers, who often have to play a new part in the relationship. Older patients, receiving pension or living off of retirement funds, do not need to worry as much anymore about providing for themselves and their family.

### Significance of age and KPS

Our regression analysis revealed that after applying several models, KPS was associated stronger with HRQoL than calendric age. This is probably not surprising. However, given the fact that the newer studies led to different treatment schemes based on age, e.g., for GBM, it seems to be debatable to assign patients to therapy schemes considering solely their age in light of our findings: deciding on a more or less aggressive treatment should be based not only on KPS or calendric age alone, but also on patient perceived HRQoL. Undoubtedly, especially in elderly HGG patients, we should include geriatric assessment what seems to be helpful in geriatric cancer patients in general [37, 38].

### Limitations and strengths of the study

Of note, we have to discuss several flaws of the study. First, we report a post hoc analysis, what gives a retrospective character to the prospectively collected data. Therefore, findings have to be interpreted carefully. Second, as already mentioned, due to the time consuming assessment, we observed a selection bias, as patients in a worse clinical condition in both groups independently of age declined participation in the study. Therefore, we report data of a well-functioning cohort which may influence mean scores, yet, since patients are assumed to have dropped out in both groups, the general findings regarding between group differences should be valid. Further, as mentioned above, the two patient populations exhibited differences regarding WHO grade, ongoing chemotherapy, and mean time since diagnosis. However, in a recent published review including glioblastoma patients, the authors found that chemotherapy and radiotherapy may not have harmful effects on HRQoL in fit elderly cancer patients [39]. A further limitation of the study is that we did not assess the comorbidities of the patients, which may have also influenced the results in the two different groups overestimating the influence of the HGG diagnosis. Furthermore, quality of life is influenced by multiple factors in this group, of which not all could be assessed as we analyzed content-driven variables to reduce multiple testing. Therefore, results and comparisons have to be interpreted carefully.

Yet, the strength of this study is that rare data are available for this patient group, and our study may on the one hand provide a basis for decision-making in daily clinical practice and on the other hand motivate to conduct further clinical studies assessing HRQoL in elderly HGG patients.

## Conclusion

Physical functioning was significantly reduced in the elderly compared with younger HGG patients. Emotional functioning and DT scores were comparable. KPS shows a greater association with HRQoL than with calendric age in HGG patients reflecting the particular importance for adequate assessment of HRQoL and general condition in elderly patients.
